# A Key Role of microRNA-29b for the Suppression of Colon Cancer Cell Migration by American Ginseng

**DOI:** 10.1371/journal.pone.0075034

**Published:** 2013-10-09

**Authors:** Deepak Poudyal, Xiangli Cui, Phuong Mai Le, Anne B. Hofseth, Anthony Windust, Mitzi Nagarkatti, Prakash S. Nagarkatti, Aaron J. Schetter, Curtis C. Harris, Lorne J. Hofseth

**Affiliations:** 1 Department of Drug Discovery and Biomedical Sciences, South Carolina College of Pharmacy, University of South Carolina, Columbia, South Carolina, United States of America; 2 Shanxi Medical University, Shanxi, China; 3 Institute for National Measurement Standards, National Research Council, Ottawa, Canada; 4 School of Medicine, University of South Carolina, Columbia, South Carolina, United States of America; 5 Laboratory of Human Carcinogenesis, Center for Cancer Research, National Cancer Institute, National Institutes of Health, Bethesda, Maryland, United States of America; Winship Cancer Institute of Emory University, United States of America

## Abstract

Metastasis of colon cancer cells increases the risk of colon cancer mortality. We have recently shown that American ginseng prevents colon cancer, and a Hexane extract of American Ginseng (HAG) has particularly potent anti-inflammatory and anti-cancer properties. Dysregulated microRNA (miR) expression has been observed in several disease conditions including colon cancer. Using global miR expression profiling, we observed increased miR-29b in colon cancer cells following exposure to HAG. Since miR-29b plays a role in regulating the migration of cancer cells, we hypothesized that HAG induces miR-29b expression to target matrix metalloproteinase-2 (MMP-2) thereby suppressing the migration of colon cancer cells. Results are consistent with this hypothesis. Our study supports the understanding that targeting MMP-2 by miR-29b is a mechanism by which HAG suppresses the migration of colon cancer cells.

## Introduction

Colorectal Cancer (CRC) is the third most commonly diagnosed cancer in both men and women and the third leading cause of cancer death. In the USA, the American Cancer Society estimated 141,210 new cases of colorectal cancer and 49,380 deaths in 2011. Metastasis leads to 90% of cancer-related mortalities [1], [2]. In principle, during metastasis of CRC, some cancer cells from the primary tumor mass invade surrounding tissue, intravasate into the vasculature to travel through blood and lymphatic vessels, arrest in distant capillaries, extravasate into parenchyma of distant tissue (primarily liver and lungs) where they seed new colonies to form the macroscopic secondary tumors [3]. These metastatic cancer cells lose their ability to adhere to neighboring tumor cells and develop migratory and invasive properties to disseminate to distant metastatic organs. While doing so, the metastatic cells undergo changes in gene expression and function, thereby gaining more mesenchymal- like features and this process is termed as Epithelial to Mesenchymal Transition (EMT), a crucial event in malignancy. MicroRNAs (MiRs) are small non-coding RNAs of approximately 22 nucleotides (nts) long that post-transcriptionally regulates the gene expression in plants and animals. In animals, miRs target transcripts through imperfect base pairing of 2-7 nts of 5′-end of miR (so-called ‘seed’ sequence) to multiple sites in 3′-untranslated regions (UTRs) of target mRNA, and this imperfect miR-mRNA hybrids with central bulges (nt 9–12) recruits miRNP (microRNA Ribonucleoprotein complex) that enable translational inhibition or exonucleolytic mRNA decay [Reviewed in [4]]. Many housekeeping genes have evolved with shorter length of 3′-UTR to avoid miR regulation [5]. About 50% of annotated human miR genes are located in cancer associated genomic regions or fragile sites that are susceptible to amplification, deletion and translocation in variety of tumors including colon tumors [6]. Because of this some miRs could act either as tumor suppressor or oncogenes [7], [8], [9], [10], [11]. Expression profiling analysis has revealed characteristic miR signatures that can predict the clinical outcomes of CRC [12], [13].

One of the classical hallmarks of cancer is the ability for tumor cells to invade and metastasize [14]. miRs are both positive and negative regulators of cancer metastasis [15], [16], [17]. One negative regulator of cancer metastasis is miR-29b [For example [18], [19], [20], [21], [22]]. miR-29b belongs to the miR-29 family. The miR-29 family is comprised by three paralogs: miR-29a, -29b and -29c. miR-29a and miR-29b1 are located on chromosome 7q32; miR-29b2 and miR-29c are located on chromosome 1q23 [23]. miR-29b1 and miR-29b2 sequences are identical but are distinguished b1 and b2 due to difference in locus. MMP-2, an extracellular matrix (ECM) degrading enzyme that has a major implication in metastasis and angiogenesis has been shown to be the direct target of miR-29b [20].

American ginseng (AG; *Panax quinquefolius*) is an obligate shade perennial native of North America. From Bioassay guided fractionation of AG, we have recently shown that a Hexane fraction of AG (HAG) is a potent anti-oxidant and anti-cancer agent [24], [25]. To date, only limited anti-cancer studies of lipophilic extracts of AG have been carried out, and these studies are mostly focused on anti-proliferative and cytotoxic effects [26], [27], [28], [29]. To further understand the anti-cancer mechanism of HAG (a lipophilic extract), we studied the role of miR in cancer cell migration.

##  Materials and Methods

### Hexane Fraction of AG

The *P. quinquefolius* extract has been described previously in detail by our laboratory [30]. As well, we have recently described the generation of the HAG used in the present study [24].

### Cell Cultures

HCT 116 wild-type (WT), LOVO and DLD-1 colon cancer cells were purchased from American Type Culture Collection (ATCC; Manassas, VA). HCT 116 cells were cultured in McCoy's medium (ATCC, Manassas, VA); LOVO cells were culture in F-12K medium (ATCC, Manassas, VA); and DLD-1 cells were culture in RPMI-1640 medium. All media was supplemented with 10% Newborn Calf Serum (NBCS; GIBCO/Life Technologies, Grand Island, NY), 2 mM glutamine (Biofluids, Rockville, MD), penicillin (10 U/ml, Biofluids) and streptomycin (10 μg/ml, Biofluids).

### Global mir Expression

HCT 116 WT cells were seeded at 1×10^6^ cells/plate in 6 well plates in four replicates. After culture for 24 h, 260 µg/mL HAG was added into each well. Cells were harvested at 0, 12, and 24 h separately in RNase free EP tubes. Total RNA was extracted using TRIzol reagent (Ambion, Austin, TX). RNA concentration was determined by the Nanodrop 2000 (NanoDrop, Wilmington, DE). 100 ng of RNA from HCT 116 WT cells was used for the nCounter miRNA Expression Assay v1.2 (Nanostring Technologies, Seattle, WA) containing 800 miRNA's following the manufacturer's instructions.

### miR-29b Expression

Cells were seeded, exposed to vehicle (media only) or 260 µg/ml HAG, and harvested at 24 hr. For miR-29b detection, 10 ng of total RNA was used to reverse-transcribe to cDNA using TaqMan miR Reverse Transcription kit (Applied Biosystems, Foster City, CA) according to manufacturer's instructions and miR primers specific for hsa-miR-29b for detection and the small nuclear protein RNU6B (U6) for normalization (Applied Biosystems). qPCR measurement of miR-29b and U6 expression was performed using TaqMan miR Assays (Applied Biosystems) with the 7300 PCR Assay System (Applied Bioystems). The comparative threshold cycle (Ct) method was used to evaluate the relative abundance of miR-29b compared with U6 expression (fold changes relative to U6). All experimental treatments were carried out on three separate occasions; each time with three replicates.

### siRNA and miR Transfection

For MMP-2 siRNA, 1.5×10^5^ cells were grown in medium in 6 well plates 1 day before transfection. Using INTERFERin siRNA Transfection Reagent (Plyplus, iLllkirch, France), the cells were transfected with 5 nM of MMP-2 Trilencer-27 human siRNA (Origene, Rockville, MD). After 48 h of transfection, the cells were processed for western blot analysis to evaluate the efficacy of knock down (Figure S3). For MirVana-miR-29b inhibitor and mirVana-Negative control inhibitor (Ambion, Austin, TX) transfection, 1.5×10^5^ cells were grown in medium in 6 well plates 1 day before transfection. Using INTERFERin siRNA Transfection Reagent (Polyplus Transfections, Illkirch, France), the cells were transfected with 10 nmol/L of MirVana-miR-29b inhibitor or MirVana-Negative control inhibitor. After 48 h of transfection, the cells were harvested in RNase free EP tubes. Total RNA was extracted using TRIzol regent. RNA concentration was determined by Nanodrop 2000. 10 ng of total RNA was reverse-transcribed to cDNA using TaqMan MicroRNA Reverse Transcription kit with microRNA primers specific for hsa-miR-29b and the small nuclear protein RNU6B (U6) for normalization. qPCR measurement of miRNA-29b and U6 expression was performed using TaqMan MicroRNA Assays with the 7300 PCR Assay System. The relative fold change in miR-29b level was used to represent the relative abundance of miRNA-29b compared with U6 expression. As per the supplier of mirVana-miR-29b Inhibitor (Ambion by Life Technologies, Austin, TX), the efficacy with which the mammalian cells are transfected with mirVana-miR inhibitor depends on the cell type and transfection agent used. It is recommended that optimization experiment (Concentrations from 1 nM to 100 nM miR inhibitor) be carried out to obtain the maximum activity with minimum cytotoxicity. The transfection efficacy and the origin of 3 cell lines HCT116, LoVo and DLD-1 are different and from the optimization experiment (data not shown), mirVana miR-29b Inhibitor showed maximum activity at 10 nM after 48 h of transfection for HCT116 cells and 50 nM for LoVo and DLD-1 cells. After 48 h of transfection with MirVana-miR-29b Inhibitor, HAG (260 µg/mL) was added for 24 h and cells were harvested for target gene (MMP-2) expression and for migration assay. All experimental control samples were treated with an equal concentration of a non-targeting inhibitor negative control sequence, for use as controls for non-sequence-specific effects in miR experiments. Mock-transfected controls did not produce any significant effect on any of the parameters analyzed.

### mRNA Analysis

Total RNA was extracted using Trizol reagent (Invitrogen, CA). One μg of total RNA served as template for single strand cDNA synthesis in a reaction using oligo(dT) primers and AMV reverse transcriptase (Promega Corp, WI) under conditions indicated by the manufacturer. PCR of cDNA samples was performed with samples amplified for 30 cycles of denaturation at 94°C for 30 s, annealing at 50°C for 30 s, and extension at 72°C for 30 s with final extension at 72°C for 10 min. The sequences for Real Time PCR primers used were: MMP-1 Forward 5′-AGG TCT CTG AGG GTC AAG CA-3′; MMP-1 Reverse 5′-CTG GTT GAA AAG CAT GAG CA-3′; MMP-2 Forward 5′-ACA TCA AGG GCA TTC AGG AG-3′; MMP-2 Reverse 5′-GCC TCG TAT ACC GCA TCA AT-3'; MMP-7 Forward 5′-GAG TGC CAG ATG TTG CAG AA-3′; MMP-7 Reverse 5′-AAA TGC AGG GGG ATC TCT TT-3′; MMP-9 Forward 5′-TTG ACA GCG ACA AGA AGT GG-3′; MMP-9 Reverse 5'-GCC ATT CAC GTC GTC CTT AT-3′ and GAPDH Forward 5′-GAG TCA ACG GAT TTG GTC GT-3′, GAPDH Reverse 5′-TTG ATT TTG GAG GGA TCT CG-3′ (Integrated DNA Technologies, Inc). Real-time PCR (qPCR) was performed using the 7300 Real-Time PCR Assay System (Applied Biosystems, CA) with Power SYBR green PCR master mix (Applied Biosystems, CA) and primers for MMP-1, −2, −7, −9 and GAPDH according to the vendor's protocol. The MMP gene expression was normalized by GAPDH gene expression. The fold change in the gene expression is relative to the vector treated [1x Phosphate Buffered Saline (PBS)] cells harvested at 24 h.

### Western Blot Analysis and Antibodies

Western blots were carried out as described previously [31]. Antibodies used include: MMP-2 (Rabbit polyclonal, diluted 1 in 500, cat #4022s; Cell Signaling Technology, Danvers, MA), and GAPDH (Rabbit polyclonal, diluted 1 in 1000, cat #5174; Cell Signaling Technology, Danvers, MA). For all the blots, a standard protein (BenchMark Prestained Protein Ladder; Invitrogen, Carlsbad CA) was run to ensure the correct molecular weight of each bands observed. Horseradish peroxidase-conjugated anti-rabbit secondary antibodies were purchased from Amersham Biosciences (Piscataway, NJ). Secondary antibodies were diluted at 1∶2000. All antibodies were diluted in 5% milk/PBST (0.1% Tween 20 in 1× PBS). Western blot signal was detected by Pierce ECL Western Blotting Substrate (Thermo Scientific, Rockford, IL) and developed onto Hyperfilm.

### *In Vitro* Assay For Migration

The 24 well Costar transwell permeable support with a 8-μm pore size polycarbonate membrane (Corning Incorporated, NY), and with a 6.5 mm insert was used to analyze the migration of tumor cells. For this migration assay, the transwell membrane was soaked with 15 μg/ml of Collagen Type I (BD Biosciences) for 30 min at 37°C in Serum free media. Collagen was removed by pipetting and collagen coated membrane was place on a 24 well plate. Cells were serum starved for 12 h and 50,000 cells were resuspended in 200 μl of Serum Free Medium (SFM) and transferred on the top chamber of the transwell. The bottom chamber was filled with 750 μl of SFM or Complete growth medium (10% NCS supplemented, as a chemoattractant for cells) or HAG (260 μg/ml in complete medium). The transwell plate was incubated at 37°C for 12 h. After incubation, medium was removed from the chamber, the transwell membrane was washed in 1X PBS and cells were fixed by formaldehyde (3.7% in PBS) and permeabilized by 100% methanol and stained with 0.4% Crystal violet stain. The membrane was washed with 1X PBS and the non-migrated on the top of the transwell membrane were scraped off with the sterile cotton swab. The transwell membrane was cut out from the transwell chamber and fixed on microscopic slide with permount and viewed under the microscope (100x magnification). 7 random sections were photographed from each slide and the migrated cells on the bottom of the transwell membrane were automatically counted by using Image J software (http://rsbweb.nih.gov/ij/).

### Statistical Analysis

For global miR analysis, all data were imported into NSolver Analysis Software v1.0 (Nanostring Technologies) and normalized to the geometric mean of the 100 miRs with the highest expression values. Normalized data was imported into BRB-ArrayTools v4.1.0 for analysis. Prior to analysis, data was filtered where any value less than 10 was omitted and any miR missing in >50% of samples were excluded leaving 248 miR's for analysis. The filtering criteria was set such that any normalized data point <10 or the log2 transformed data value <3.321928 were called missing and were excluded because that is at the level of background. Only those data points >10 or log2 transformed data value >3.321928 were taken into consideration and passed the filtering criteria leaving 248 miRs for analysis. Class comparison test utilized Student's T-tests to compare miR's of treated vs. untreated cells. Trend tests used linear regression modeling on ordered categorical variables of 0 h, 12 h and 24 h. The Benjamini-Hochberg procedure was used to calculate false discovery rates. When more than two groups were compared, we determined statistical differences using a one-way analysis of variance, followed by a Scheffe's multiple comparison test. If two groups were compared, we used a Student's T-test. The P value chosen for significance in this study was 0.05.

## Results

### HAG Induces miR-29b In Colon Cancer Cells

**Figure 1:**
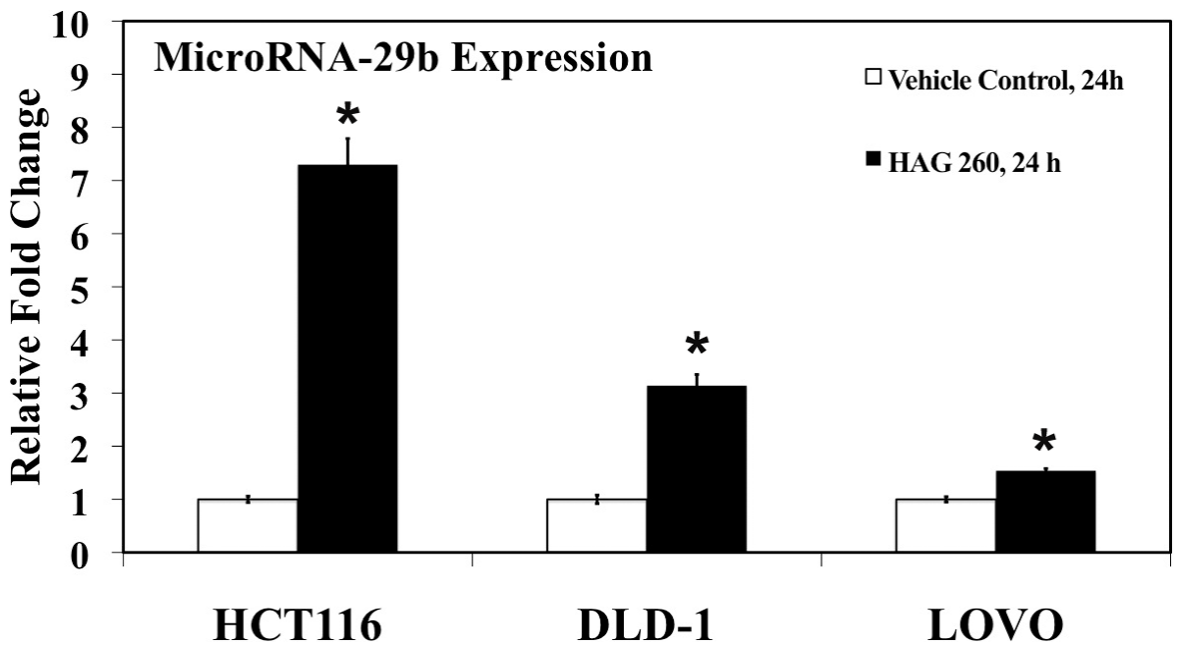
miRNA-29b expression increases in colon cancer cells after exposure to Hexane fraction of American Ginseng (HAG). HCT 116, DLD-1, and LOVO cells were exposed to 260 µg/mL HAG for 24 h (n = 3 per time point). Relative endogenous miR-29b expression levels were detected by qRT-PCR using Taqman primers and probes to detect mature miR-29b and the small nuclear RNA RNU6B (U6), an internal control. Relative miR-29b expression levels were normalized to the average value of the non-treated samples (0 h). *, indicates significant difference (pvalue <0.005) from the 0 h control.

American Ginseng and HAG have both exerted preventive effect on the chemically induced colon cancer model [24], [32]. To initiate our study, since miRs have been shown to have both pro-tumor and anti-tumor properties, we looked at the effect of HAG on global miR expression in colon cancer cells. There was a significant positive correlation in 2 miRs, and significant negative correlation in 6 miRs after exposure of HCT 116 cells to HAG (260 µg/ml) for 0 h, 12 h, and 24 h (Table 1). Based on the understanding that miR-29 family has been down regulated in multiple malignancies [21], [23], [33], [34], that several studies have shown that up regulation of miR-29 to have anti-tumor effects [22], [35], and that increased expression of miR-29b was shown with two different statistical methods (Tables 1 and 2), we focused on this miR. To confirm miR-29b up-regulation by HAG, we repeated the experiment and examined miR-29b upregulation by qRT-PCR. Consistent with the global miRNA analysis results, Figure 1 shows that there was increased miR-29b expression (7.3-fold) with exposure of HCT 116 cells to HAG. There was also an increase in miR-29b expression with exposure of two other colon cancer cell lines (DLD-1, 3.1-fold; and LOVO, 1.5-fold) (Figure 1).

**Table 1:** MicroRNA expression changes with exposure to HAG (260 μg/ml): Trend Change Analysis.

	Correlation coefficient	Parametric p-value	FDR*	Unique id
1	−0.899	0.0008802	0.218	hsa-miR-938
2	−0.739	0.0081706	0.958	hsa-miR-203
3	−0.719	0.0241946	0.958	hsa-miR-1975
4	0.621	0.0348001	0.958	hsa-miR-29b
5	−0.621	0.0348001	0.958	hsa-miR-600
6	−0.688	0.0350915	0.958	hsa-miR-1244
7	−0.695	0.0441186	0.958	hsa-miR-548o
8	0.592	0.0457531	0.958	hsa-miR-590-5p

**Table 2:** MicroRNA expression changes with exposure to HAG (260 μg/ml): Fold Change Analysis (Untreated Vs Treated).

	Parametric p-value	FDR*	Geom mean of intensities in treated	Geom mean of intensities in untreated	Fold-change	Unique id
1	0.0043164	0.737	72.78	44.07	1.65	hsa-miR-1308
2	0.0071632	0.737	45.92	30.11	1.53	hsa-miR-151-3p
3	0.0089105	0.737	40.62	54.78	0.74	hsa-miR-203
4	0.0245673	0.945	66.4	35.04	1.9	hsa-miR-590-5p
5	0.0317838	0.945	22.76	36.21	0.63	hsa-miR-1975
6	0.0434628	0.945	182.05	216.73	0.84	hsa-miR-222
7	0.0446853	0.945	14.08	20.47	0.69	hsa-miR-34c-3p
8	0.0448605	0.945	14.52	21.68	0.67	hsa-miR-142-3p
9	0.0449166	0.945	16.15	28.51	0.57	hsa-miR-548o
10	0.0457623	0.945	11.65	17.72	0.66	hsa-miR-938
11	0.0470418	0.945	67.08	48.86	1.37	hsa-miR-29b

* , Indicates False Discovery Rate.

### HAG Suppresses MMP-2 Expression In Colon Cancer Cells

**Figure 2:**
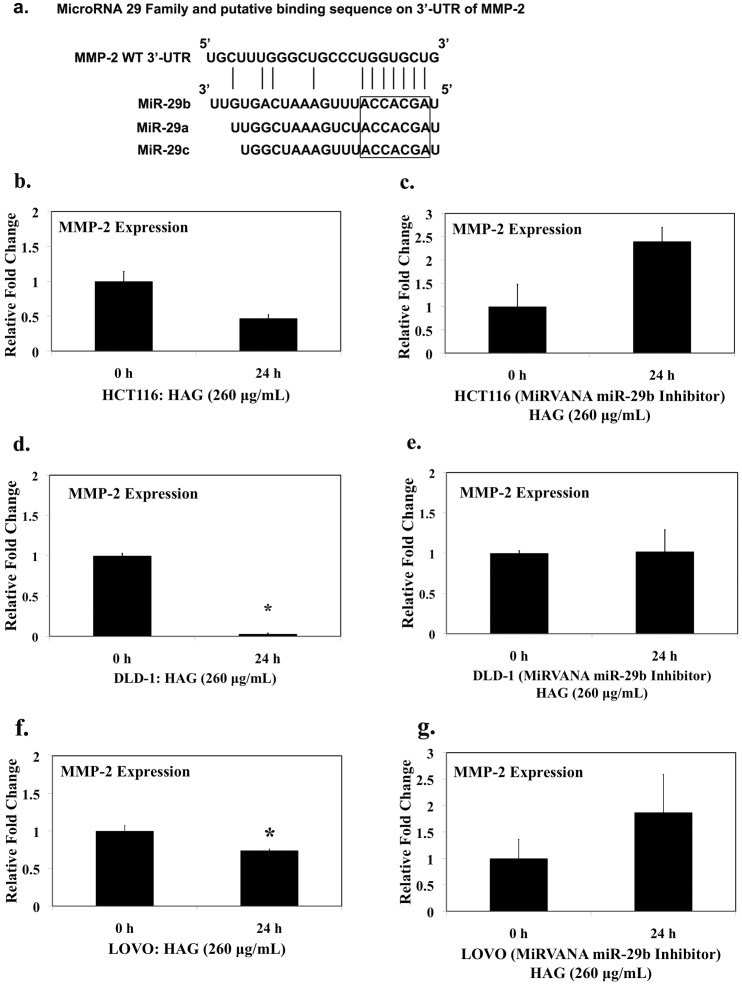
Hexane fraction of American Ginseng (HAG) suppresses MMP-2 gene expression. (A) miR-29 family (miR-29a/b/c) and its putative binding sequence in the 3'-UTR of MMP-2 gene. The seed sequence of miR-29 family is shown in the box. (B) HCT116 WT cells were exposed to 260 µg/mL HAG for 24 h. (C) HCT116 cells transfected with 10 nM of mirVANA miR-29b, 48 h and exposed to 260 µg/mL HAG for 24 h. (D) DLD-1 cells were exposed to 260 µg/mL HAG for 24 h. (E) DLD-1 cells transfected with 50 nM of mirVANA miR-29b, 48 h and exposed to 260 µg/mL HAG for 24 h. (F) LOVO cells were exposed to 260 µg/mL HAG for 24 h. (G) LOVO cells transfected with 50 nM of mirVANA miR-29b, 48 h and exposed to 260 µg/mL HAG for 24 h. Relative MMP-2 expression level was detected by qRT-PCR. MMP-2 mRNA for each sample was normalized by GAPDH expression. Fold change in the MMP-2 mRNA level was relative of non-treated cells harvested at 0 h (n = 3 per time point). Results indicate the Hexane Fraction of AG suppresses MMP-2 mRNA level compared to the non-treated cells. *, Indicates significant difference, P value <0.05 from 0 h control.

Potential target genes of miR-29b were first predicted using online databases, including miRTarBase, TargetScan, PicTar, and miRanda. MMP-2 was predicted to be the potential target of miR-29b by all these databases (Figure 2A). Because MMP-2 is overexpressed in tumor tissues [36], [37], [38], and has a direct implication in metastasis and angiogenesis of cancer cells [39], [40], we first examined the effects of HAG on MMP-2 expression. Treatment of HCT-116 cells with HAG for 24 h resulted in the reduction of MMP-2 gene expression by approximately half fold [(1±0.14 to 0.57±0.05), p-value  = 0.078] (Figure 2B). HAG treatment for 24 h, significantly reduced the expression of MMP-2 gene in DLD-1 cells [(1±0.03 to 0.03±0.01), p-value <0.005] (Figure 2D). Similarly treatment of LOVO cells with HAG for 24h resulted in the reduction of MMP-2 expression [(1±0.06 to 0.74±0.017), p-value <0.05] (Figure 2F). To verify if HAG reduces the MMP-2 activity, HCT-116 cells were treated with HAG for 24h and MMP-2 protein was analyzed. Figure S4, HAG reduced the pro- and active- MMP-2 protein expression; confirming that HAG reduced MMP-2 activity and gene expression. Additionally other ECM degrading MMPs, such as MMP-1, MMP-7 and MMP-9 gene expression was not regulated by HAG treatment (Table S2). Interestingly, these MMPs (MMP−1, −7 and −9) are not the direct target of miR-29b.

Further, to verify if the HAG mediated suppression of MMP-2 gene is dependent on miR-29b, we silenced miR-29b (Figure S1) using a miR-29b inhibitor (see methods 4.2.5). HAG does not suppress MMP-2 gene expression when miR-29b is silenced (Figures 2C, 2E and 2G). In fact, there is a 2.4 fold increase in expression of MMP-2 in miR-29b silenced HCT116 cells when exposed to HAG, 24 h (Figure 2C). There was no decrease in MMP-2 gene expression in miR-29b silenced DLD-1 and LOVO colon cancer cells when exposed to HAG, 24 h (Figures 2E and 2G). All together, these results are consistent with the hypothesis that the suppression of MMP-2 by HAG is dependent on miR-29b.

### HAG Suppresses Migration of Colon Cancer Cells

**Figure 3:**
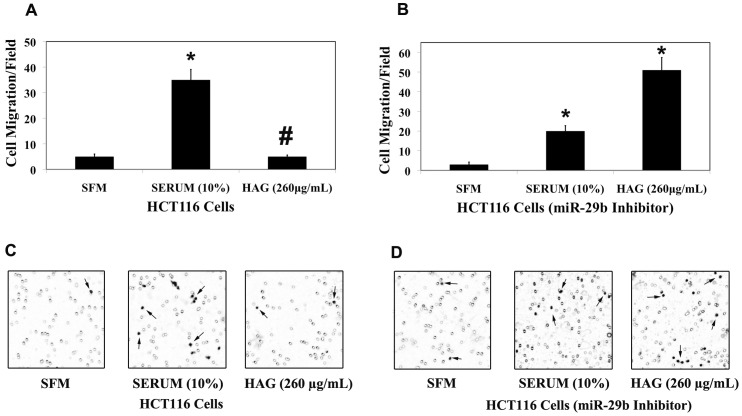
Hexane fraction of American Ginseng (HAG) represses HCT116 colon cancer cell migration *in vitro.* Collagen type-I (15 µg/mL) coated transwell chamber were applied with 5×10^4^ HCT116 cells (A/B) for 12 h. The lower chamber contains SFM/Serum (10%) or HAG (260 µg/mL in complete medium). (A) 5×10^4^ HCT116 WT cells were applied to the upper chamber of the transwell membrane. (B) 5×10^4^ HCT116 (transfected with mirvana miR-29b, 10 nM, 48 h) cells were applied to the upper chamber of transwell membrane. After 12 h incubation at 37°C, the cells migrated to the inside (lower membrane) of transwell membrane was counted using ImageJ software (7 random microscopic fields (100X) were evaluated for cell counting). (C) Depicts the representative picture of HCT116 WT cell migration from each treatment. (D) Depicts the representative picture of HCT116 (transfected with mirvana miR-29b, 10 nM, 48 h) cell migration from each treatment. The background in the picture shows the 8 μm pore in the transwell membrane. *, indicates significant difference (pvalue <0.005) when compared to SFM. #, indicates significant difference (pvalue <0.005) when compared to 10% Serum.

**Figure 4:**
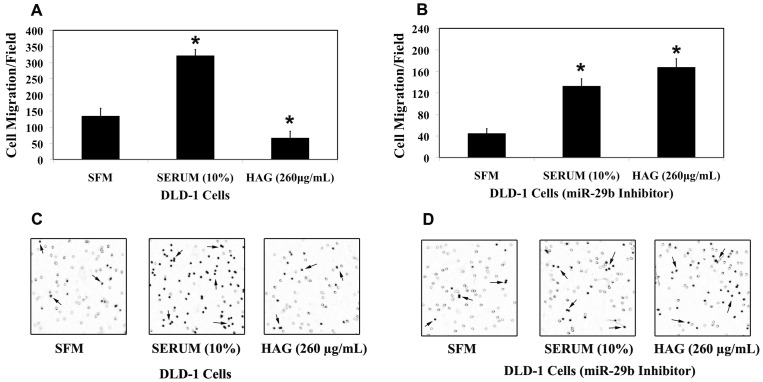
Hexane fraction of American Ginseng (HAG) represses DLD-1 colon cancer cell migration *in vitro.* Collagen type-I (15 µg/mL) coated transwell chamber were applied with 5×10^4^ DLD-1 cells (A/B) for 12 h. The lower chamber contains SFM/Serum (10%) or HAG (260 µg/mL in complete medium). (A) 5×10^4^ DLD-1 WT cells were applied to the upper chamber of the transwell membrane. (B) 5×10^4^ DLD-1 (transfected with mirvana miR-29b, 10 nM, 48 h) cells were applied to the upper chamber of transwell membrane. After 12 h incubation at 37°C, the cells migrated to the inside (lower membrane) of transwell membrane was counted using ImageJ software (7 random microscopic fields (100X) were evaluated for cell counting). (C) Depicts the representative picture of DLD-1 WT cell migration from each treatment. (D) Depicts the representative picture of DLD-1 (transfected with mirvana miR-29b, 10 nM, 48h) cell migration from each treatment. The background in the picture shows the 8 μm pore in the transwell membrane. *, indicates significant difference (pvalue <0.005) when compared to SFM.

MiR-29b targets key players to repress invasion and metastasis [19], [20], [21], [22]. MMP-2 is involved in the migration of colon cancer cells (Table S1; Figure S2). About 3.5-fold reduction in the number of cells migrated/microscopic field in the HCT116 cells MMP-2 knock/down cells compared to the HCT116 WT cells in the presence of 10% Serum as chemoattractant is reported (Table S1; Figure S2). This is a clear indication that MMP-2 is a key factor in the regulation of cell migration, however it should not be ruled out that other ECM degrading MMPs such as MMP-1, MMP-7, MMP-9 and MMP-13 have been shown be associated with Colorectal cancer progression [37], [41], [42], [43], [44]. Since HAG up-regulates miR-29b expression and down-regulates the ECM degrading enzyme MMP-2 gene expression, we further investigated the functional effect of HAG on cancer cell migration. In HCT116 cells, HAG suppressed the migration of cancer cells by almost 7-fold (Figures 3A and 3C). In the miR-29b silenced HCT-116 cells, HAG didn't suppress migration. Consistent with MMP-2 results (Figure 2C), in the absence of miR-29b activity, HAG elevated the number of cells migrating to the lower chamber of transwell membrane by almost 2.5 fold when compared to positive control (Figures 3B and 3D). Similarly, HAG suppressed the migration of DLD-1 cells only in the presence of miR-29b, where it reduced the number of migrating cells by almost 5 fold compared to the positive control (Figures 4A and 4C). HAG did not exert anti-migratory activity when miR-29b was silenced in DLD-1 cells (Figures 4B and 4D). Overall, results here show miR-29b is at the crossroads in the ability of HAG to exert anti-migratory activities.

## Discussion

Here we have demonstrated that HAG suppresses migration of colon cancer cell. This anti-metastatic property of HAG is mediated by the up-regulation of microRNA-29b, which directly targets and down-regulates a key molecule involved in metastasis, MMP-2.

Global miR analysis of HAG – treated HCT116 cells resulted in the elevated expression of miR-29b that matched both the trend and fold-change statistical analysis (Tables 1 and 2). There were 8 miRNAs (hsa-miR-938, hsa-miR-203, hsa-miR-1975, hsa-miR-29b, hsa-miR-600, hsa-miR-1244, hsa-miR-548o and hsa-miR-590-5p) that were statistically (p<0.05) up or down-regulated. A positive correlation coefficient indicated increased levels of miR with increased exposure to HAG (0 h to 12 h to 24 h) with only 2 miRs (hsa-miR-29b and hsa-miR-590-5p) falling in this category. Of these 2 miRs, miR-29b had the highest correlation coefficient value of 0.621. As well, since miR-29b was also statistically significantly up-regulated by HAG in the fold change analysis (Table 2), and that this miR has been shown by others to play a tumor suppressor role [23], [33], [45], [46], [47], we focused on miR-29b for this particular study. The role of miR-29b as a tumor suppressor has been well elucidated in several malignancies including, AML [23], [45], lung [33], CLL [46], [47] and cholangiocarcinoma [48]. Down-regulation of the miR-29 family has been reported in several human cancers including lung [49], prostate [50], invasive breast cancer [51]. Recently, Kuo et'al have reported a lower expression level of miR-29a/c in a colorectal cancer early recurrence group compared with that of a non-early recurrence group indicating low level expression of miR-29a/c as a potential biomarker for early recurrence of CRC [52]. Our results have better elucidated the possible mechanisms of this finding.

The miR-29 family consists of three members: miR-29a, miR-29b, and miR-29c (miR29a/b/c) that display high sequence similarity and share a common seed sequence for target recognition (Figure 2A). Others have reported an inverse relationship regarding the expression of miR-29b and MMP-2 [20], [22], [53], [54]. As well, because miR databases (miRTarBase, TargetScan, PicTar, and miRanda) indicate MMP-2 as a potential target of miR-29b, we examined the functional significance of this. MMP-2, also known as gelatinase A or type IV collagenase, is an ECM degrading enzyme, and is widely expressed in most tissues and cells [55]. As well, MMP-2 is overexpressed in tumor tissues [36], [37], [38] and activation of MMP-2 results in ECM degradation, which facilitates the invasion and metastasis of tumor cells [56]. Our real-time RT-PCR data showed that HAG reduces the MMP-2 expression by 2-fold in HCT116 cells and to approximately 30-fold reduction in DLD-1 cells (Figures 2C and 2E). HAG also reduced MMP-2 protein expression (Figure S4). When miR-29b activity is silenced by transfecting colon cancer cells with a miRVana miR-29b Inhibitor (Figure S1), HAG has no effect on reduction of MMP-2 expression (Figures 2C, 2E and 2G) and to some extent induced the MMP-2 expression. The reason could be due to the absence of endogenous miR-29b, HAG was unable to regulate miR-29b that further enhanced the MMP-2 secretion. Some of the components present in the HAG, could have the potential to enhance MMP-2 expression directly in the absence of endogenous miR-29b, however this needs further study to be confirmed. Therefore a vigorous isolation process of active components of HAG is underway and future studies regarding this are in the line. All together, our data suggest that miR-29b is critical for HAG to suppress the MMP-2 expression in cancer cells.

Matrix metalloproteinases have been regarded as key molecules assisting tumor cells during metastasis [57], [58], [59], [60]. The MMPs are a family of zinc-containing endopeptidases best known for their roles in physiological and pathological remodeling of the ECM during angiogenesis, wound healing, embryogenesis, tumor metastasis, and various cardiovascular and inflammatory diseases [61], [62]. It has been shown that miR-29b is involved in the negative regulation of metastasis in several cancer types [18], [20], [63]. Combining these findings, where miR-29b is a negative regulator of metastasis and targets key player of metastasis MMP-2, and HAG induces miR-29b and suppresses MMP-2 expression (Tables 1 and 2; Figures 1, 2 and S4), we asked the question if HAG functionally suppresses metastasis of colon cancer cells. We demonstrated that HAG functionally suppresses the *in vitro* metastasis of colon cancer cells by performing migration assay of colon cancer cells (Figures 3A, 3C, 4A, and 4C). In the absence of miR-29b activity, HAG was not efficient in suppressing the migration of colon cancer cells (Figures 3B, 3D, 4B, and 4D). Although, yet to be shown *in vivo*, all together, our *in vitro* data indicate that HAG performs its anti-metastatic activity by regulating miR-29b.

Major components present in the Hexane extract (lipophilic extract) of AG are polyacetylenes (Panaxydiol, panaxydol and panaxynol), which comprises about 50% of the total extract, as well as fatty acids, with almost no ginsenosides [24]. We have recently shown that HAG possesses anti-inflammatory and anti-cancer properties [24]. Several other studies on anti-inflammatory and anti-cancer properties of American ginseng have focused on the ginsenoside or the saponin content of ginseng [64], [65], [66], [67], which is obtained from an aqueous ethanol extract (polar solvent) of ginseng. Of particular interest regarding our study, several studies have reported the anti-angiogenic and anti-metastatic properties of ginseng components [68], [69], [70], [71]. Ginsenoside 20(R) and 20(S)-Rg3 possess an ability to inhibit lung metastasis of tumor cells such as B16-BL6 melanoma and colon 26M3.1 by inhibition of adhesion and invasion of tumor cells and also by anti-angiogenesis activity [68]. Ginsenoside Rb2 inhibits invasion via MMP-2 suppression resulting in the inhibition of secondary spreading of uterine endometrial cancer [69]. Rd has been shown to inhibit migration of liver cancer cell lines HepG2 by reducing expression of MMP-1, MMP-2 and MMP-7 [70]. In contrast, ginsenoside Rg1 has been reported to promote and enhance angiogenesis and migration [72], [73], [74]. Relatively very few studies on cancer and inflammation are focused on the non-polar or lipophilic extract of AG, such as that found in our HAG. Consistent with our present study, however, Park et al have shown that glucocorticoid receptor-induced suppression of MMP-9 by panaxadiol and panaxatriol appears to reduce invasion of highly metastatic human fibrosarcoma cell line, HT1080 [75].

In conclusion, given the increasing understanding that ginseng and/or its' components have potent anti-cancer and anti-metastatic activities, it is important to better understand the mechanisms. Here, we have demonstrated that miR-29b and MMP-2 are key players in the ability of HAG to suppress colon cancer cell migration. Although the *in vivo* translation remains to be shown, we have shown that HAG suppresses colon cancer in mice [24], and these current experiments shed light into the mechanism by which HAG works. Our mechanistic findings open up the possibility that HAG alone, or in concert with and/or miRNA-29b mimics, may have efficacy in the chemoprevention and/or treatment of colon cancer.

## Supporting Information

Figure S1 **Suppression of endogenous microRNA-29b using mirVANA miR-29b Inhibitors.** Relative fold change in miR-29b expression normalized by endogenous control U6 snRNA after 48h of miR-29b inhibitors or Control negative oligonucleotides. (A) Relative miR-29b expression in HCT116 cells after transfection with either miR-29b inhibitor or control negative oligonucleotides (10 nM concentrations) for 48 h. (B) Relative miR-29b expression in DLD-1 and LOVO cells after transfection with miR-29b inhibitor (50 nM concentration) for 48h. *, indicates significant difference (pvalue<0.05) from the wild type control. #, indicates significant difference (pvalue<0.005) from the wild type control.
(TIF)

Figure S2 **MMP-2 is the key factor in the migration of HCT-116 cells.** Collagen type-I (15 µg/mL) coated transwell chamber were applied with 5×10^4^ HCT116 cells or MMP-2 k/d HCT116 cells for 12 h. The lower chamber contains SFM or Complete medium (10% Serum). 5×10^4^ HCT116 WT or HCT116 MMP-2 k/d cells were applied to the upper chamber of the transwell membrane. After 12 h incubation at 37°C, the cells migrated to the inside (lower membrane) of transwell membrane was counted using ImageJ software (7 random microscopic fields (100X) were evaluated for cell counting). Representative picture for each treatment is shown.
(TIF)

Figure S3 **siRNA mediated MMP-2 knock/down in HCT116 cells.** HCT116 cells were transfected with MMP-2 trilencer 27-human siRNA (0.1 nM to 10 nM). 48 h after transfection, cells were harvested and MMP-2 protein was analyzed by western blot. 5 nM MMP-2 siRNA showed maximum efficacy in silencing MMP-2.
(TIF)

Figure S4 **Hexane fraction of American Ginseng (HAG) suppresses MMP-2 activity.** HCT-116 cells were treated with 260 μg/ml of HAG for 0 and 24 h. Cells were harvested and MMP-2 activity was accessed by western blot analysis. HAG suppressed the pro- and active-MMP2 enzyme.
(TIF)
