# Supplementary figures and images for: A Key Role of microRNA-29b for the Suppression of Colon Cancer Cell Migration by American Ginseng
Source: PLoS One. 2013 Oct 9;8(10):e75034. doi: 10.1371/journal.pone.0075034 (PMC3794036; doi:10.1371/journal.pone.0075034)

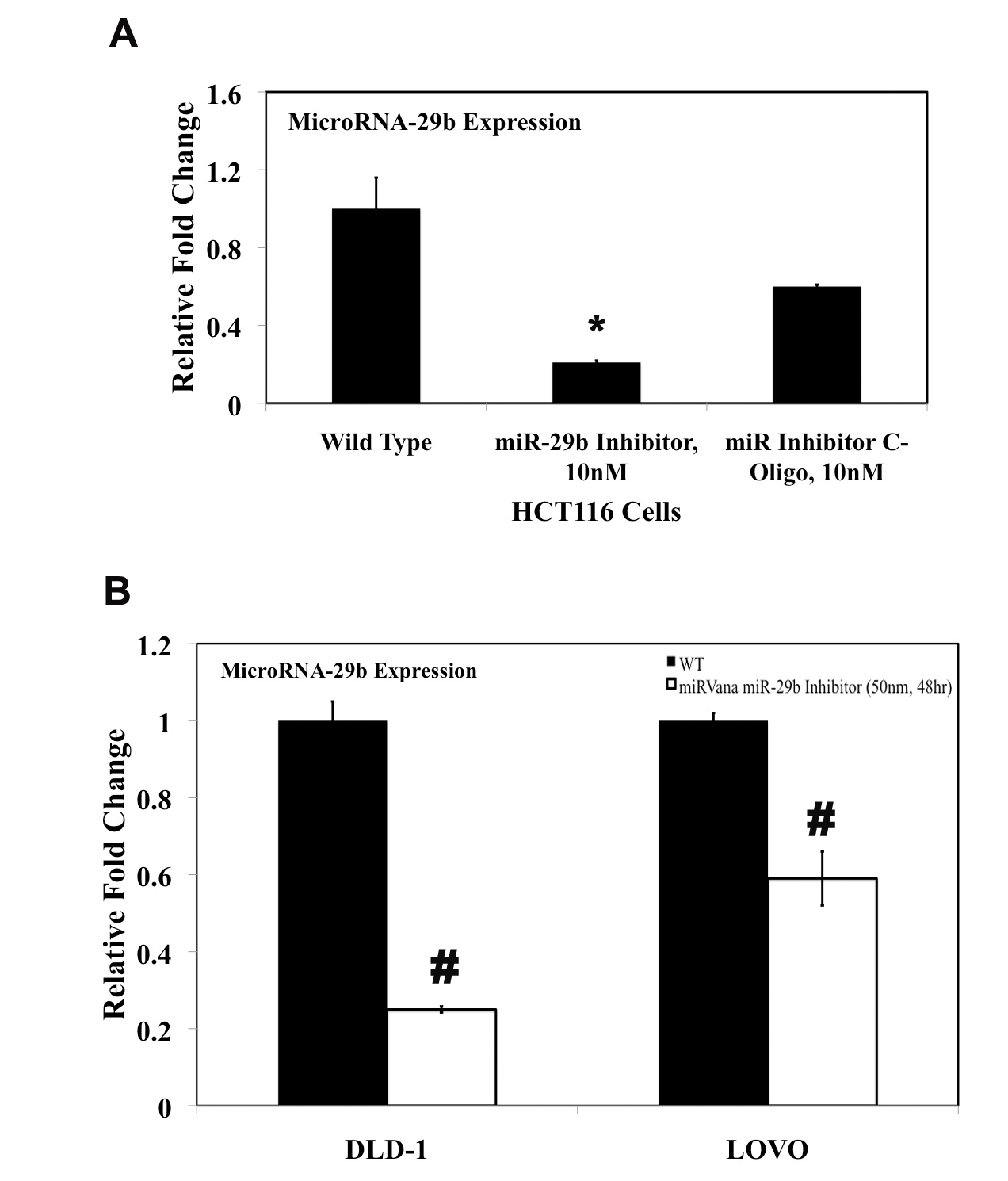

Supplement: Figure S1 — Suppression of endogenous microRNA-29b using mirVANA miR-29b Inhibitors. Relative fold change in miR-29b expression normalized by endogenous control U6 snRNA after 48h of miR-29b inhibitors or Control negative oligonucleotides. (A) Relative miR-29b expression in HCT116 cells after transfection with either miR-29b inhibitor or control negative oligonucleotides (10 nM concentrations) for 48 h. (B) Relative miR-29b expression in DLD-1 and LOVO cells after transfection with miR-29b inhibitor (50 nM concentration) for 48h. *, indicates significant difference (pvalue<0.05) from the wild type control. #, indicates significant difference (pvalue<0.005) from the wild type control. (TIF) [file pone.0075034.s001.tif]

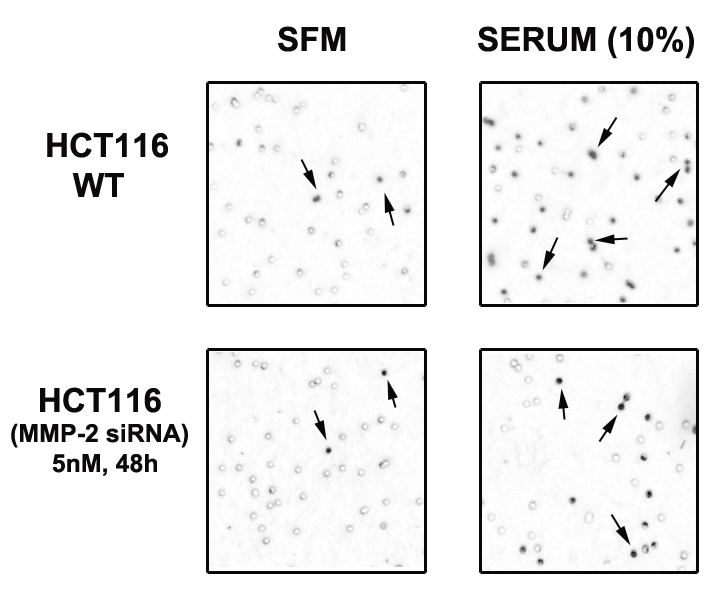

Supplement: Figure S2 — MMP-2 is the key factor in the migration of HCT-116 cells. Collagen type-I (15 µg/mL) coated transwell chamber were applied with 5×10^4^ HCT116 cells or MMP-2 k/d HCT116 cells for 12 h. The lower chamber contains SFM or Complete medium (10% Serum). 5×10^4^ HCT116 WT or HCT116 MMP-2 k/d cells were applied to the upper chamber of the transwell membrane. After 12 h incubation at 37°C, the cells migrated to the inside (lower membrane) of transwell membrane was counted using ImageJ software (7 random microscopic fields (100X) were evaluated for cell counting). Representative picture for each treatment is shown. (TIF) [file pone.0075034.s002.tif]

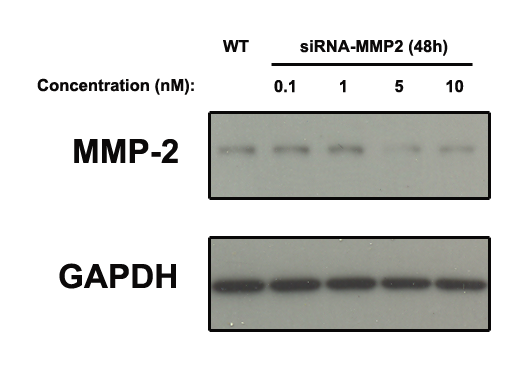

Supplement: Figure S3 — siRNA mediated MMP-2 knock/down in HCT116 cells. HCT116 cells were transfected with MMP-2 trilencer 27-human siRNA (0.1 nM to 10 nM). 48 h after transfection, cells were harvested and MMP-2 protein was analyzed by western blot. 5 nM MMP-2 siRNA showed maximum efficacy in silencing MMP-2. (TIF) [file pone.0075034.s003.tif]

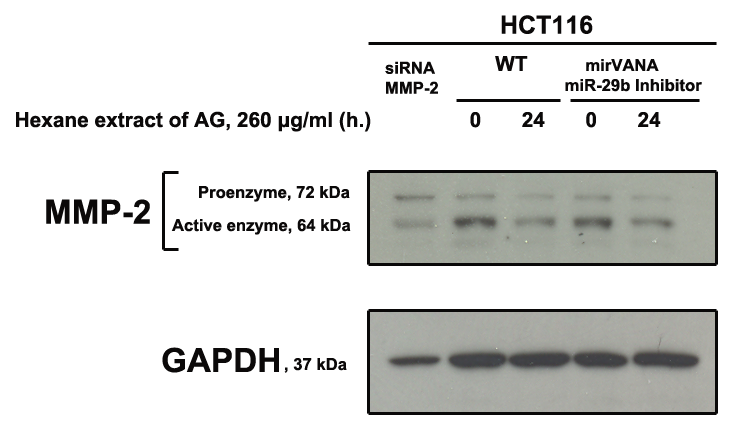

Supplement: Figure S4 — Hexane fraction of American Ginseng (HAG) suppresses MMP-2 activity. HCT-116 cells were treated with 260 μg/ml of HAG for 0 and 24 h. Cells were harvested and MMP-2 activity was accessed by western blot analysis. HAG suppressed the pro- and active-MMP2 enzyme. (TIF) [file pone.0075034.s004.tif]
